# The Identification of a Single-Base Mutation in the Maize *Dwarf 1* Gene Responsible for Reduced Plant Height in the Mutant 16N125

**DOI:** 10.3390/plants14081217

**Published:** 2025-04-15

**Authors:** Ping Wang, Bingbing Liang, Zhengjun Li, Huaiyu Dong, Lixia Zhang, Xiaochun Lu

**Affiliations:** 1 Institute of Plant Protection, Liaoning Academy of Agricultural Sciences, Shenyang 110161, China; pingw-556@163.com (P.W.);; 2 Institute of Sorghum, Liaoning Academy of Agricultural Sciences, Shenyang 110161, China

**Keywords:** maize, plant heigh, *D1* gene, dwarf mutant, genetic mapping, breeding

## Abstract

Maize (*Zea mays* L.) is a globally vital crop for food, feed, and biofuel production, with plant height (PH) being a key agronomic trait that significantly influences yield, lodging resistance, and stress tolerance. This study identified a single-base mutation in the *D1* (*Dwarf 1*) gene responsible for the dwarf phenotype in the maize mutant 16N125. Through genetic analysis and fine mapping, the candidate region was localized to chromosome 3, narrowing it down to an interval containing three genes. Sequencing revealed a non-synonymous mutation in *D1*, which encodes a gibberellin 3-beta-dioxygenase, leading to amino acid substitutions at positions 61 and 123. Genetic analysis of F2 populations confirmed that the mutation at position 61 was responsible for the dwarf trait. Furthermore, the mutation was detected in several Chinese inbred lines, indicating its potential role in dwarfing under specific conditions. These findings provide critical insights into the genetic mechanisms regulating maize plant height, offering valuable information for breeding programs focused on improving crop architecture and yield to address the challenges of global food security and climate change.

## 1. Introduction

Maize (*Zea mays* L.) is one of the most important global crops serving as a staple food, feed, and biofuel source. It is a cornerstone of global agriculture, providing essential nutrients and energy for both humans and livestock. Over the past century, maize production has increased eightfold, largely due to genetic improvements, advanced agricultural practices, and the development of high-yielding hybrids [1,2,3]. However, with the growing global population, which is projected to reach nearly 10 billion by 2050, and the challenges posed by climate change, there is an urgent need to further enhance maize productivity, resilience, and adaptability [4,5]. Among the various agronomic traits that influence maize yield and performance, plant height (PH) stands out as a critical factor [6,7]. Plant height serves as a crucial morphological trait that profoundly influences stress tolerance in plants. Taller plants exhibit superior light capture efficiency, particularly in dense canopies, thereby enhancing their photosynthetic capacity under light-limiting conditions. This architectural advantage also extends to thermal regulation, as their vertical profile enables exposure to varied microclimates that facilitate heat dissipation during high-temperature stress. Furthermore, shorter plants enhance lodging resistance through improved stem strength and a lower center of gravity, reducing yield losses under mechanical stress [6,7,8,9,10].

Plant height is a complex trait influenced by a combination of genetic, environmental, and hormonal factors. It is closely associated with other important traits such as ear height, internode length, and node number, which collectively contribute to biomass production and yield potential [11,12]. In energy maize, taller plants correlate strongly with a higher biomass, ideal for bioenergy sources like cellulosic ethanol. However, in grain maize, excessive height risks lodging, reducing yield and harvest efficiency. Breeding must thus balance biomass production and structural strength based on end-use goals [13].

The importance of plant height in crop improvement was highlighted during the Green Revolution of the mid-20th century, when the discovery and utilization of semi-dwarf genes (*rht1* in wheat and *sd1* in rice) revolutionized cereal production. These semi-dwarf varieties exhibited improved lodging resistance, increased yield, and enhanced responsiveness to fertilizers, leading to significant gains in global food production [14,15,16]. In maize, however, the genetic mechanisms controlling plant height are more complex, and identifying the genes that regulate this trait has proven to be more challenging. Nevertheless, the potential benefits of optimizing plant height in maize are immense, as it could lead to the development of high-yielding, stress-resistant varieties that are better suited to the changing climatic conditions and the increasing demand for food and bioenergy [17].

To date, over 50 genes have been implicated in the regulation of maize plant height, many of which are involved in the biosynthesis, transport, and signaling pathways of key plant hormones such as gibberellins (GAs), abscisic acid, and brassinosteroids [18,19,20,21]. Gibberellins, in particular, play a central role in regulating plant growth and development, including stem elongation, leaf expansion, and flowering. Mutations in genes involved in gibberellin biosynthesis or signaling often result in dwarf phenotypes, as seen in the well-studied *dwarf1* (*D1*) gene, which encodes a gibberellin 3-beta-dioxygenase [22,23,24,25]. Other genes, such as *ZmBr2* and *ZmGA2ox3*, have also been shown to influence plant height by modulating hormone levels and cell elongation [19,26].

Despite these advances, the genetic mechanisms underlying the natural variation in maize plant height remain poorly understood, primarily due to the complexity of the trait and the limited number of successfully cloned genes. Traditional linkage mapping and quantitative trait locus (QTL) analysis have identified numerous genomic regions associated with plant height, but the resolution of these methods is often insufficient for precise gene identification [27,28]. Moreover, the interaction between multiple genes and environmental factors further complicates the genetic dissection of plant height. In recent years, however, advances in genomics and sequencing technologies have provided new tools for studying complex traits in crops. One such tool is bulked segregant analysis combined with next-generation sequencing (BSA-seq), which has emerged as a powerful method for rapidly identifying candidate genes associated with specific traits [29,30,31,32]. This approach significantly reduces the number of samples required for genotyping and focuses on individuals with extreme phenotypes, making it particularly suitable for studying complex traits such as plant height.

In this study, we employed BSA-seq to identify a novel mutation in the *D1* gene responsible for the dwarf phenotype in the maize mutant 16N125. The *D1* gene, also known as ZmGA3ox2, is a key regulator of gibberellin biosynthesis and has been associated with several dwarfing alleles in maize [21,22,23,24,25]. By combining BSA-seq with fine mapping and genetic analysis, we were able to narrow down the candidate region to an interval on chromosome 3 and identify a non-synonymous mutation in the *D1* gene that leads to amino acid substitutions at positions 61 and 123. Genetic analysis of F2 populations confirmed that the mutation at position 61 was responsible for the dwarf trait also found in several Chinese inbred lines, suggesting its potential role in dwarfing under specific conditions.

We aimed to identify the gene responsible for the dwarf phenotype in the maize mutant 16N125 through genetic analysis and BSA-seq. We crossed 16N125 with different maize varieties to investigate the inheritance pattern of the dwarf trait. BSA-seq and fine mapping were then employed to pinpoint the candidate gene. Furthermore, we examined the presence of the identified mutation in Chinese inbred lines to explore its potential role in maize breeding. This research is expected to contribute to a better understanding of the genetic mechanisms underlying maize plant height and provide practical guidance for future maize breeding programs. By uncovering the genetic basis of plant height in maize, we can develop more efficient breeding strategies to meet the growing demands for food, feed, and fuel, while also enhancing the resilience of maize crops in the face of a changing climate.

## 2. Results

### 2.1. Phenotyping and Genetic Analysis of Dwarf Maize Plants

**Figure 1:**
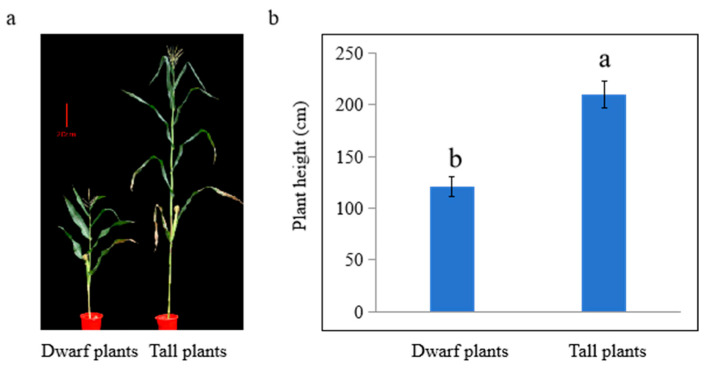
Plant height statistics of F2 generation plants. (**a**) Representative photographs of tall and dwarf plants in F2 generation; (**b**) statistical analysis of plant height. Values are means ± SD (*n* = 10). Columns marked with different letters (a–b) indicate significant differences, analyzed by SPSS software version 22.0 (Duncan’s multiple range test, α = 0.05). Bar = 20 cm.

In the summer of 2015, a mutant plant exhibiting extremely reduced stature was identified in an experimental field planted with B73, representing a naturally occurring mutation. If the naturally mutated gene can be mapped, it may represent a novel allele influencing maize plant height. Consequently, we retained this serendipitously obtained mutant and propagated it continuously for seven generations until the traits stabilized. This mutant was named 16N125 to identify the gene influencing plant height. We conducted a series of crosses between this mutant and multiple maize varieties and successfully obtained F1 generation seeds. In the following spring, these F1 seeds were sown in Shenyang city (42° N,123° E), and no significant changes in plant height or panicle length were observed, indicating a phenotype similar to that of the tall parental generation. Subsequently, after self-pollinating the F1 plants, the F2 generation was generated, and upon sowing the F2 seeds, clear trait segregation was evident. Some plants displayed a marked reduction in both plant height and panicle length. However, the segregation ratio of the F2 generation exhibited a discernible pattern solely when crossed with maize variety A101—a tall, inbred line selected and bred from America. Statistical analysis revealed that the dwarf maize plants had an average height of approximately 120 cm and the tall ones were 210 cm, confirming that the dwarf trait was completely recessive to the tall trait (Figure 1). Additionally, the numbers of tall and short plants are summarized in Table 1. According to the law of segregation, in F2 populations, the ratio of individuals with dominant traits to those with recessive homozygous traits should be 3:1. Among the 825 F2 individuals, 609 displayed a tall plant height while 216 showed a dwarf plant height. When subjected to the Chi-square test, the segregation ratio was found to be 3:1 (χ^2^ = 0.62 < 3.84, and the *p* value was greater than 0.05). The fact that χ^2^ < 3.84 indicates a significant correlation between the theoretical segregation ratio and the actual one. These results thus demonstrated that a single recessive gene should be responsible for controlling the dwarf trait.

### 2.2. BSA-Seq Analysis and Mapping of Candidate Gene

To identify novel genes influencing plant height, we constructed a population for bulked segregant analysis sequencing (BSA-seq). Fifty plants exhibiting extreme phenotypes were selected to form either the extremely tall or dwarf pools (Table 2). Using the Illumina HiSeq platform, we obtained approximately 2.2 billion raw reads from sequencing the parental plants and extreme bulks (Table 2). Following stringent quality control procedures, including adapter trimming and low-quality read removal, we retained 330.2 billion high-quality clean bases.

The quality assessment demonstrated an excellent sequencing performance, with GC content ranging from 46.31% to 46.95%. The data quality metrics exceeded standard thresholds, exhibiting Q20 ≥ 96.99% and Q30 ≥ 92.01% (Table 2). These results confirm the high reliability of our sequencing data for subsequent genetic analyses.

**Figure 2:**
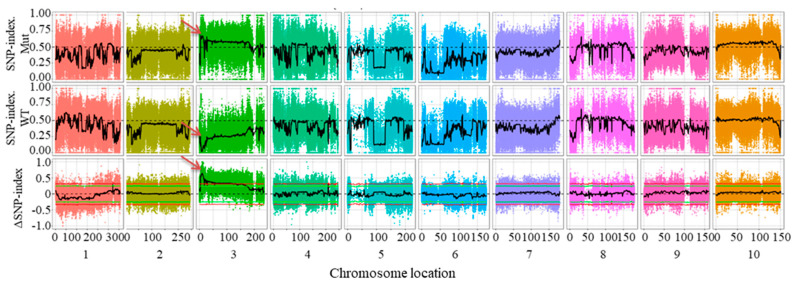
The genome-wide association mapping of plant height. The candidate SNPs and InDels when using the ΔSNP−index algorithm with a cutoff of ΔSNP−index > 0.5. The arrow indicates the region where the predicted candidate gene was located.

Single-nucleotide polymorphism (SNP) detection and filtering were performed using the GATK software toolkit (version 4.1.8.1). To identify genomic regions associated with plant height, we applied the ΔSNP-index method to assess allele frequency differences in SNPs and InDels between the two extreme bulk pools. Association intervals were determined based on fitted ΔSNP-index values, with a 99% confidence threshold (red line, Figure 2). The significant regions are summarized in Table 3 and marked by arrows in Figure 2. The most robust association was detected within a 5.6 Mb interval (7,000,000–12,600,000 bp) on chromosome 3.

### 2.3. Fine Mapping of Candidate Gene

**Figure 3:**
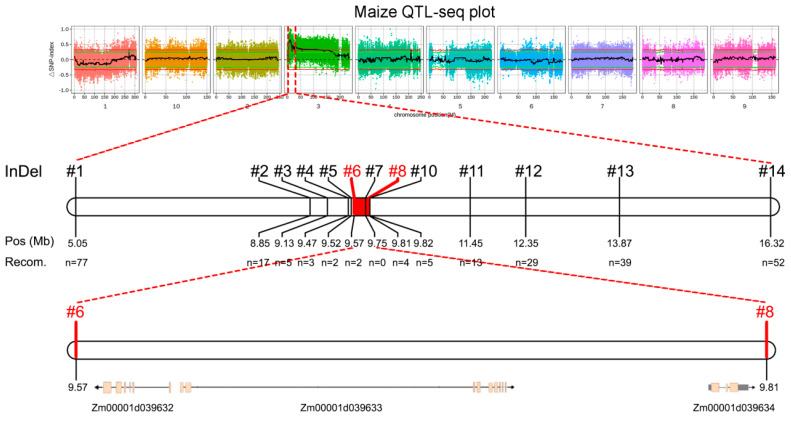
The fine mapping of the candidate gene, narrowing the candidate region through a recombinant-derived progeny testing strategy. The genetic linkage map of the candidate region of Chr.3 and the genes and diagnostic markers in the 11.27 Mb interval.

Based on the above BSA-seq results, 14 InDel markers were developed on chromosome 3, which covered a slightly larger region than predicted and displayed stable polymorphisms between the parental line and the F2 generation individuals whose parents were 16N125 and A101 (Figure 3). By using a recombinant-derived progeny testing strategy [33,34], we screened 752 F2 plants and identified 248 individuals recombinant between the markers InDel-1 and InDel-14, as exhibited in Figure 3.

Through successive [33,34] screenings, we identified 38 recombinants, narrowing the candidate region to the interval between markers InDel-2 and InDel-10. To further refine the location, we developed six additional InDel markers and screened for rare recombinants. The final two recombinants delimited the critical region to a 240 kb segment (9.57–9.81 Mbp) containing only three annotated genes (Figure 3, Table 4). Notably, *D1*, a strong candidate gene, was located within this interval.

### 2.4. Identification of Candidate Gene Related to Plant Height in 16N125 Plants

**Figure 4:**
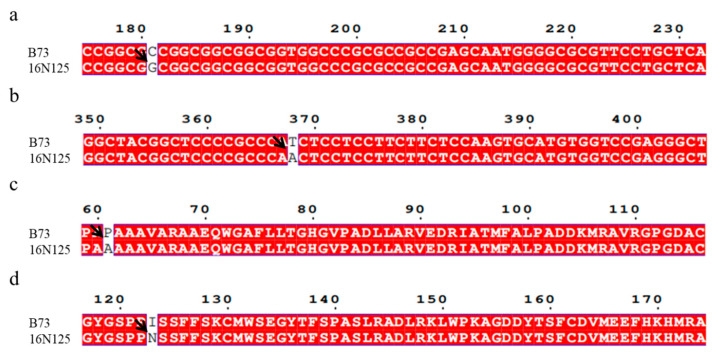
CDS and amino acid sequence analysis of *D1* genes from different cultivars. CDSs (**a**,**b**) and amino acid (**c**,**d**) sequences of *D1* arranged to show variations. Multiple sequence alignment performed by espript 3.0 online tool (https://espript.ibcp.fr/ESPript/cgi-bin/ESPript.cgi (accessed on 4 December 2024)). Arrows point to locations of variations in sequences.

To pinpoint the causal gene, we conducted Sanger sequencing for all three candidate genes in both parental lines. While nucleotide variations were present in all three genes, only *D1* exhibited a non-synonymous mutation in the amino acid sequence of 16N125 plants (Figure 4). For further analysis, we aligned and compared the coding sequences (CDSs; Figure 4a,b) and corresponding amino acid sequences (Figure 4c,d) of *D1* to illustrate the functional impact of this mutation. In contrast to B37, the amino acid sequence of 16N125 contained a substitution of alanine for proline at position 61 (Figure 4c) and asparagine for isoleucine at position 123 (Figure 4d), which was caused by the conversion of “C” to “G” at position 181 (Figure 4a) and “T” to “A” at position 368 (Figure 4b) in the CDS, respectively. Interestingly, the two substitutions in 16N125 were located at the sites corresponding to *d1-4*, a previously reported allele of *d1* that contains a large sequence deletion. The mutation sites in 16N125 plants were found within the regions of these missing fragments. Therefore, we concluded that the mutation in the *D1* gene might be responsible for the dwarfing phenotype observed in the 16N125 plants. To determine which of the two mutation sites played a role in the dwarfing of the 16N125 plants, we examined the sequence of the *D1* gene using Sanger sequencing in some randomly selected extremely tall and extremely dwarf plants from the F2 generation, and the results are listed in the Table S1. Among the eight dwarfing progenies, seven exhibited a homozygous G at position 181, identical to that of 16N125. In contrast, the tall progenies either possessed a C at position 181, similar to A101, or were heterozygous with C/G at this position. The phenotype resulting from this gene mutation aligns with the characteristics of a recessive gene. Notably, while the dwarf plants showed consistency with 16N125 at position 368, no discernible pattern emerged in the gene sequence of the tall offsprings. Interestingly, five tall plants were homozygous for allele A at this locus, which is consistent with the genotype of the dwarf parent 16N125. In summary, the mutation at position 61, rather than the other one, plays a regulatory role in maize plant height.

### 2.5. Investigation of D1 Mutant Genes in Widely Utilized Chinese Inbred Lines

**Figure 5:**
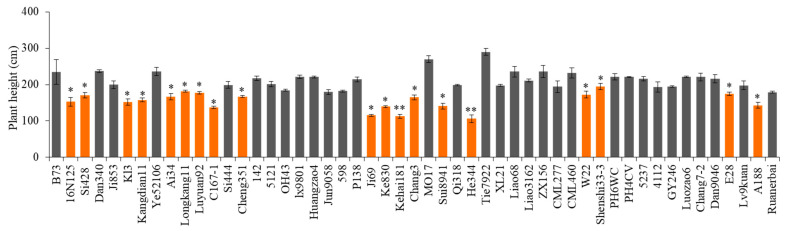
Plant height statistics of 50 commonly used inbred lines and 16N125. Plant height investigated (*n* ≥ 15) in 2022. Asterisks indicate statistically significant differences compared with B73 using Student’s *t*-test (*, *p* < 0.05; **, *p* < 0.01).

To investigate the amino acid mutation sites of the *D1* gene in 16N125 plants within a natural population, we sowed 50 commonly available Chinese inbred lines, including B73, in 2023. We then analyzed the variation in the *D1* gene among these lines relative to B73 using Sanger sequencing. Additionally, plant height was measured at maturity. As shown in Figure 5, 18 inbred lines exhibited a significant reduction in plant height compared to B73. Notably, the amino acid sequences of the *D1* gene in Ke830, Kehai181, Chang3, and Sui8941 were identical to those of B73, suggesting that the reduced height in these four lines may be attributed to other dwarfing genes or factors (Supplementary File S1).

The results above indicate a substitution from proline to alanine at position 61 of the 16N125 amino acid sequence. Similarly, several inbred lines exhibiting reduced plant height also possessed alanine at this position, including Si428, Longkang11, Luyuan92, W22, E28, and A188. Additionally, glutamine was observed at position 61 in other lines, such as C167-1, Cheng351, Ji69, and He344 (Supplementary File S1). Therefore, 76.92% of the 13 inbred lines that exhibited a change in the *D1* amino acid sequence and a corresponding decrease in plant height demonstrated a mutation at amino acid position 61. The cause of their dwarfism remains unclear. Additionally, all inbred lines exhibiting a height more significant than that of B73 possessed proline at amino acid position 61, which is consistent with the residue found in B73. Based on these findings, it can be inferred that the 61st amino acid of the maize *D1* gene may influence plant height under specific conditions. However, the mutation at the 123rd amino acid position in the *D1* gene of 16N125 appears to be an isolated event, as no asparagine was detected at this position in other natural populations. Other mutations in the *D1* gene of various inbred lines compared to B73 are summarized in Supplementary File S1, but none were found to be significantly associated with changes in plant height. The variations in the *D1* gene for the remaining lines are detailed in Supplementary File S1. Notably, only three inbred lines—Kl3, Kangdian11, and Ai34—displayed a dwarfing phenotype despite retaining proline at position 61. The experiment was repeated three times, with similar results obtained.

## 3. Discussion

In the dynamic field of maize research, the exploration of genetic mechanisms underlying important traits has been significantly advanced by the advent of high-throughput sequencing technologies, with BSA-seq being a prominent example. In recent years, numerous studies have capitalized on BSA-seq to dissect the genetic architecture of various maize traits. Yu et al. (2024) employed BSA-seq to map chromosomal regions associated with low-temperature tolerance during maize germination [35]. Their research not only identified potential candidate genes but also provided valuable insights into the complex genetic network governing cold stress response in maize. Similarly, Zheng et al. (2023) combined BSA-seq and RNA-seq to identify genes associated with the visual stay-green phenotype during the maize maturation stage [36]. These studies not only showcase the power of BSA-seq but also lay a solid foundation for our investigation into the genetic basis of maize.

Plant height critically affects stress tolerance. Taller plants capture more light in dense canopies, improving photosynthesis under low light. Their height also aids heat dissipation by accessing cooler air layers. Conversely, shorter plants resist lodging better due to their stronger stems and lower center of gravity, protecting yield under mechanical stress [6,7,8,9,10]. In energy maize, taller plants mean more biomass for bioenergy. But in grain maize, too much height causes lodging, cutting yield. Breeding needs to balance biomass and strength for different uses [13].

Our study focused on identifying the gene responsible for the dwarf phenotype in the maize mutant 16N125. Through a comprehensive approach involving genetic analysis and BSA-seq, we successfully narrowed down the candidate gene region to chromosome 3. Subsequent fine-mapping and Sanger sequencing revealed a non-synonymous mutation in the *D1* gene, which encodes a gibberellin 3-beta-dioxygenase, as the cause of the dwarfing effect in 16N125. This finding aligns with the well-established role of the *D1* gene in regulating plant height through its involvement in the gibberellin biosynthesis pathway [22,23,25].

To date, four distinct alleles of the *D1* gene (*d1-3286*, *d1-6039*, *d1-4*, and d1-6016) have been identified [25]. The d1-3286 allele has a 4.5 kb Copia-type retrotransposon inserted at nucleotide position 69 of the *ZmGA3ox2* gene [25]. This insertion disrupts the normal transcriptional and translational processes of the gene, leading to a significant reduction in the production of bioactive gibberellins and ultimately resulting in a dwarf phenotype. The *d1-6039* allele, on the other hand, has a single-nucleotide deletion at position 399 within the first exon [25]. This deletion causes a frameshift mutation, which leads to the premature termination of translation at the 163rd amino acid, severely impairing the function of the encoded enzyme and causing dwarfism. The *d1-6016* allele features a large deletion spanning 2301 bp, encompassing 508 bp of upstream regulatory sequences and 1793 bp of the *ZmGA3ox2* coding region [25]. Such a large-scale deletion disrupts both the regulatory and coding functions of the gene, resulting in a profound dwarf phenotype. The *d1-4* allele contains a 487 bp deletion affecting 389 bp of the first exon and 98 bp of the first intron [25]. This deletion also affects the proper splicing and expression of the gene, leading to a reduction in the activity of the gibberellin 3-beta-dioxygenase enzyme and dwarf plant growth.

In contrast to these previously reported alleles, the 16N125 mutant exhibits a unique mutation pattern. It has a substitution of alanine for proline at position 61 and asparagine for isoleucine at position 123 in the amino acid sequence of the *D1* gene. Through genotypic analysis of the F2 generation and an in-depth investigation of 50 Chinese inbred lines, we determined that the mutation at position 61 is likely the key factor regulating plant height. This is a novel finding, as previous studies on the *D1* gene have not reported such a mutation at this specific position.

This newly identified mutation in 16N125 adds a new dimension to our understanding of the genetic diversity of the *D1* gene. By incorporating this mutation into the *d1-4* allele, we propose an additional locus that contributes to the dwarfing effect of the *d1-4* mutation. This discovery has far-reaching implications for understanding the intricate mechanisms of plant height regulation in maize. It provides a new perspective on how mutations in the *D1* gene can disrupt the gibberellin biosynthesis pathway and ultimately affect plant growth and development.

From a practical perspective, this finding offers potential genetic resources for maize breeding programs. Dwarf or semi-dwarf maize varieties with improved lodging resistance are highly desirable in modern agriculture. Lodging is a major problem in maize production, especially in regions prone to strong winds and heavy rains. By developing varieties with optimized plant heights through the manipulation of the *D1* gene, breeders can enhance the lodging resistance of maize crops, ensuring more stable yields. For example, in regions with high-wind climates, semi-dwarf maize varieties can better withstand strong gusts, reducing the risk of plant lodging and subsequent yield losses [37].

Maize has a long history of cultivation in China since its introduction in the 16th century [38]. Over time, a rich and diverse collection of germplasm resources has been established. However, prior to our study, the status of dwarf genes within common Chinese germplasm resources was not well characterized. By systematically investigating *D1* mutations in 50 widely utilized Chinese inbred lines through Sanger sequencing, we have filled this knowledge gap. Our results indicate that the 61st amino acid of the maize *D1* gene may play a crucial role in influencing plant height under specific conditions.

This information is invaluable for maize breeders in China. It allows them to better understand the genetic background of local inbred lines, which is essential for rational parent selection in breeding programs. For instance, breeders can now identify inbred lines with favorable *D1* gene mutations and use them as parents to develop new varieties with improved plant architecture. By selecting parents with the appropriate *D1* gene mutations, breeders can potentially develop maize varieties that are not only more resistant to lodging but also have enhanced yield potential. In addition, understanding the genetic diversity of the *D1* gene in Chinese inbred lines can help breeders develop varieties that are better adapted to local environmental conditions, such as different soil types and climate patterns [39].

Furthermore, our study also has implications for future research on maize genetics. The identification of this novel mutation in the *D1* gene provides a starting point for further functional studies. For example, researchers can investigate the physiological and biochemical effects of this mutation by analyzing hormone levels, gene expression patterns, and plant growth responses in different genetic backgrounds. Additionally, studying the interaction of the *D1* gene with other genes in the gibberellin biosynthesis pathway and related pathways can provide a more comprehensive understanding of the genetic network governing maize plant height. This knowledge can be applied to develop molecular markers for marker-assisted selection, enabling breeders to efficiently screen for desirable plant height traits in breeding programs.

In conclusion, our study not only validates the effectiveness of BSA-seq in gene mapping but also makes significant contributions to the understanding of the genetic mechanism of maize plant height. The identification of the novel mutation in the *D1* gene and its association with plant height in Chinese inbred lines provide a solid theoretical basis for future maize breeding. This research offers practical guidance for developing maize varieties with improved traits, which is crucial for meeting the growing global demand for food and ensuring sustainable agriculture in the face of climate change. By continuously exploring the genetic diversity of maize and identifying genes associated with important traits, we can develop more resilient and productive maize varieties and contribute to global food security.

## 4. Materials and Methods

### 4.1. Plant Materials, DNA Extraction, and Sanger Sequencing

To comprehensively characterize the phenotypic traits of maize, a total of fifty inbred lines and the control group plants were planted in 2020, 2021, and 2022 at the experimental station of Liaoning Academy of Agricultural Sciences located in Shenyang, Liaoning Province, China (42° N, 123° E). Each experimental plot measured 4 m in length and 2.2 m in width, with three rows planted per plot. The row spacing was maintained at 60 cm, while the plant spacing within rows was 30 cm. When it came to the phenotype evaluation process, more than 15 individual plants were carefully selected from the central part of each plot. Specifically, plant height was measured precisely from the ground level all the way up to the top of the plants when they reached the maturity stage. It is worth noting that this measurement method was implemented by referring to the approach proposed by Zou et al. [40], and was further appropriately optimized to ensure its accuracy and suitability for this particular study.

Genomic DNA was extracted following the CTAB protocol. Each sample consisted of 5–6 plants from each accession. The concentration and quality of the extracted DNA were assessed using an ND-1000 spectrophotometer (manufactured by NanoDrop, Wilmington, DE, USA) as well as through electrophoresis on 1.0% agarose gels with lambda DNA as the standard. For the amplification of fragments of *D1*, PCR was carried out in a 25 μL reaction mixture. This mixture contained 2× EXtaq Mix (produced by Takara, Kusatsu, Japan), 0.4 μM of each primer, 0.125 μg of DNA, and an appropriate amount of double-distilled water. The PCR amplification protocol was designed as follows: An initial denaturation cycle was performed at 95 °C for 4 min. Subsequently, 30 cycles were conducted, with each cycle involving denaturation at 95 °C for 30 s, annealing at 57 °C for 30 s, and extension at 72 °C for 2.5 min. The primers utilized for both Sanger sequencing and PCR amplification are presented in Table S2. The PCR products were then sequenced by Biomarker Technologies Corporation (located in Beijing, China).

### 4.2. SNP Library Construction and High-Throughput Sequencing

The specific length amplified fragment sequencing (SLAF-seq) utilized in our study was carried out with certain adaptations based on the protocol detailed by Sun et al. [41]. The construction of the SLAF library encompassed several vital steps, which were specifically engineered to boost the yield of SLAFs, cut down on repetitive SLAFs, and ensure a balanced distribution of SLAFs, thereby maximizing the sequencing efficiency.

Firstly, a pre-design experiment was implemented to determine the most favorable conditions for constructing the SLAF library. This step was aimed at elevating the overall efficiency of SLAF-seq. Subsequently, in line with the pre-determined plan, genomic DNA was incubated at 37 °C in the presence of *Mse* I (from New England Biolabs, NEB; Ipswich, MA, USA), T4 DNA ligase (NEB), ATP (NEB), and Mse I adapter. After that, the reaction mixture was heat-inactivated at 65 °C and then underwent digestion with the additional restriction enzyme *Alu* I at 37 °C.

Next, polymerase chain reactions (PCRs) were conducted using the diluted restriction–ligation samples, along with dNTPs, Taq DNA polymerase (NEB), and *Mse* I primer containing barcode 1. The PCR products were purified by employing the E.Z.N.A. Cycle Pure Kit (Omega Bio-Tek, Norcross, GA, USA) and then combined in a pool. The pooled samples were then incubated at 37 °C with *Mse* I, T4 DNA ligase, ATP, and a Solexa adapter. Purification was achieved through the use of a Quick Spin column (Qiagen, Hilden, Germany), and the samples were then separated on a 2% agarose gel. Fragments within the size range of 450–500 bp (incorporating indexes and adapters) were isolated by means of a Gel Extraction Kit (Qiagen, Hilden, Germany).

These isolated fragments were then subjected to PCR amplification with Phusion Master Mix (NEB) and Solexa Amplification Primer Mix (Illumina, Inc., San Diego, CA, USA) to integrate barcode 2, following the guidelines provided for Illumina sample preparation. The amplified products were gel-purified, by extracting the DNA fragments sized between 450 and 500 bp, and were then diluted for paired-end sequencing on the Illumina HiSeq 2000 platform (Illumina, Inc., San Diego, CA, USA) at Biomarker Technologies Corporation in Beijing (http://www.biomarker.com.cn (accessed on 7 November 2024)). Subsequently, SNP genotyping and evaluation were carried out.

All SLAF paired-end reads with clear index information were clustered based on sequence similarity, which was detected through one-to-one alignment using BLAT (−tileSize = 10; − stepSize = 5) [42]. Sequences sharing over 90% identity were grouped into one SLAF locus. By making use of the minor allele frequency (MAF) evaluation, alleles were defined within each SLAF.

In the mapping populations of maize, SLAFs having a sequence depth lower than 107 were excluded from the subsequent analysis. SLAFs with 2–4 tags were identified as polymorphic SLAFs and regarded as potential markers. Tags with a total coverage of at least 10× were retained. Sequences were mapped onto the B73 reference genome [43], and SNPs were called using the TASSEL 3.0 GBS pipeline (www.maizegenetics.net/tassel**/** (accessed on 9 January 2024)). Missing data were imputed with NPUTE [44].

## 5. Conclusions

A single-base substitution in the *D1* gene, which encodes gibberellin 3β-dioxygenase, results in recessive dwarfism in the maize mutant 16N125. Unlike the previously reported *d1* alleles, this particular missense mutation is rather subtle. It manages to strike a balance between reducing plant height and minimizing pleiotropic effects. Moreover, as discovered within the Chinese germplasm, it presents a valuable genetic resource for breeding semi-dwarf maize varieties with enhanced lodging resistance.

## Figures and Tables

**Table 1:** Phenotype analysis for tall/dwarf plant height in F2 populations.

Generation	Total Plants	Tall	Dwarf	^a^ Expected Ratio	^b^ χ^2^	^c^ *p* Value
F_2_	825	609	216	3:1	0.62	0.43

^a^ Expected ratio of dominant trait individuals–recessive trait individuals = 3:1. ^b^ χ^2^: χ^2^ < 3.84 is considered as significantly correlated. ^c^ *p* value: *p* value > 0.05 indicates no statistically significant differences.

**Table 2:** Sequencing statistics for BSA-seq samples.

Sample	Clean Reads	Clean Bases	GC (%)	Q20 (%)	Q30 (%)
A101	168,863,930	25,329,589,500	0.4631	0.9741	0.9296
16N125	172,965,252	25,944,787,800	0.4695	0.9699	0.9201
Dwarf	913,765,764	137,064,864,600	0.4653	0.9762	0.9337
Tall	945,640,034	141,846,005,100	0.4657	0.9732	0.9274
Sum	2,201,234,980	330,185,247,000	0.4659	0.9733	0.9277

**Table 3:** Statistics of relevant area information.

Chromosome ID	Start	End	Size (Mb)
3	3,800,000	17,900,000	14.1
3	18,400,000	28,000,000	9.6
3	28,700,000	55,300,000	26.6
3	55,800,000	122,100,000	66.3
3	126,300,000	131,500,000	5.2
3	132,300,000	134,200,000	1.9
3	151,300,000	152,200,000	0.9
3	152,500,000	153,200,000	0.7
3	154,200,000	154,300,000	0.1

**Table 4:** Gene description from NCBI of 3 genes in predictive region.

Gene Number	Annotation
Zm00001d039632	Putative polyol transporter 1, *Arabidopsis thaliana* (mouse-ear cress)
Zm00001d039633	Pleiotropic drug resistance protein 15, *Oryza sativa* subsp. *japonica* (rice)
Zm00001d039634	Gibberellin 3-beta-dioxygenase 2-2, *Triticum aestivum* (wheat)

Genes were blasted on NCBI website (https://www.ncbi.nlm.nih.gov/ (accessed on 4 December 2023)).

## Data Availability

The data presented in this study are available upon request from P.W.

## Supplementary Materials

The following supporting information can be downloaded at: https://www.mdpi.com/article/10.3390/plants14081217/s1, File S1: Amino acid sequence of D1 genes in 50 Chinese inbred lines; Table S1: The sanger sequencing results of the offsprings; Table S2: Primers sequences used in this study.
