# Supplementary material for: The Identification of a Single-Base Mutation in the Maize Dwarf 1 Gene Responsible for Reduced Plant Height in the Mutant 16N125
Source: Plants (Basel). 2025 Apr 15;14(8):1217. doi: 10.3390/plants14081217 (PMC12030145; doi:10.3390/plants14081217)
Supplement: Supplementary file 1 [file plants-14-01217-s001.zip › File S1. Amino acid sequence of D1 genes in 50 Chinese inbred lines.pdf]

```
              1        10        20        30        40        50
              .        .         .         .         .         .
Si428         MPTPSHLNKNPRYLDFRAARRVPESHAWPGLHDHPVVDGGAPGPDAVPVVDLGAADPA
Dan340        MPTPSHLNKNPRYLDFRAARRVPESHAWPGLHDHPVVDGGAPGPDAVPVVDLGAADPA
Ji853         MPTPSHLNKNPRYLDFRAARRVPESHAWPGLHDHPVVDGGAPGPDAVPVVDLGAADPA
KI3           MPTPSHLNKNPRYLDFRAARRVPESHAWPGLHDHPVVDGGAPGPDAVPVVDLGAADPA
Kangdian11    MPTPSHLNKNPRYLDFRAARRVPESHAWPGLHDHPVVDGGAPGPDAVPVVDLGAADPA
Ye52106       MPTPSHLNKNPRYLDFRAARRVPESHAWPGLHDHPVVDGGAPGPDAVPVVDLGAADPA
Ai34          MPTPSHLNKNPRYLDFRAARRVPESHAWPGLHDHPVVDGGAPGPDAVPVVDLGAADPA
Longkang11    MPTPSHLNKNPRYLDFRAARRVPESHAWPGLHDHPVVDGGAPGPDAVPVVDLGAADPA
Luyuan92      MPTPSHLNKNPRYLDFRAARRVPESHAWPGLHDHPVVDGGAPGPDAVPVVDLGAADPA
C167-1        MPTPSHLNKNPRYLDFRAARRVPESHAWPGLHDHPVVDGGAPGPDAVPVVDLAAADPA
Si444         MPTPSHLNKNPRYLDFRAARRVPESHAWPGLHDHPVVDGGAPGPDAVPVVDLAAADPA
Cheng351      MPTPSHLNKNPRYLDFRAARRVPESHAWPGLHDHPVVDGGAPGPDAVPVVDLAAADPA
142           MPTPSHLNKNPRYLDFRAARRVPESHAWPGLHDHPVVDGGAPGPDAVPVVDLAAADPA
5121          MPTPSHLNKNPRYLDFRAARRVPESHAWPGLHDHPVVDGGAPGPDAVPVVDLAAADPA
OH43          MPTPSHLNKNPRYLDFRAARRVPESHAWPGLHDHPVVDGGAPGPDAVPVVDLAAADPA
1x9801        MPTPSHLNKNPRYLDFRAARRVPESHAWPGLHDHPVVDGGAPGPDAVPVVDLGAADPA
Huangzao4     MPTPSHLNKNPRYLDFRAARRVPESHAWPGLHDHPVVDGGAPGPDAVPVVDLGAADPA
Jun9058       MPTPSHLNKNPRYLDFRAARRVPESHAWPGLHDHPVVDGGAPGPDAVPVVDLGAADPA
598           MPTPSHLNKNPRYLDFRAARRVPESHAWPGLHDHPVVDGGAPGPDAVPVVDLGAADPA
P138          MPTPSHLNKNPRYLDFRAARRVPESHAWPGLHDHPVVDGGAPGPDAVPVVDLGAADPA
Ji69          MPTPSHLNKNPRYLDFRAARRVPESHAWPGLHDHPVVDGGAPGPDAVPVVDLGAADPA
Ke830         MPTPSHLNKNPRYLDFRAARRVPESHAWPGLHDHPVVDGGAPGPDAVPVVDLGAADPA
Kehai181      MPTPSHLNKNPRYLDFRAARRVPESHAWPGLHDHPVVDGGAPGPDAVPVVDLGAADPA
Chang3        MPTPSHLNKNPRYLDFRAARRVPESHAWPGLHDHPVVDGGAPGPDAVPVVDLGAADPA
MO17          MPTPSHLNKNPRYLDFRAARRVPESHAWPGLHDHPVVDGGAPGPDAVPVVDLGAADPA
Sui8941       MPTPSHLNKNPRYLDFRAARRVPESHAWPGLHDHPVVDGGAPGPDAVPVVDLGAADPA
Qi318         MPTPSHLNKNPRYLDFRAARRVPESHAWPGLHDHPVVDGGAPGPDAVPVVDLGAADPA
He344         MPTPSHLNKNPRYLDFRAARRVPESHAWPGLHDHPVVDGGAPGPDAVPVVDLGAADPA
CML460        MPTPSHLNKNPRYLDFRAARRVPESHAWPGLHDHPVVDGGAPGPDAVPVVDLGAADPA
W22           MPTPSHLNKNPRYLDFRAARRVPESHAWPGLHDHPVVDGGAPGPDAVPVVDLGAADPA
PH4CV         MPTPSHLNKNPRYLDFRAARRVPESHAWPGLHDHPVVDGGAPGPDAVPVVDLGAADPA
5237          MPTPSHLNKNPRYLDFRAARRVPESHAWPGLHDHPVVDGGAPGPDAVPVVDLGAADPA
Dan9046       MPTPSHLNKNPRYLDFRAARRVPESHAWPGLHDHPVVDGGAPGPDAVPVVDLGAADPA
4112          MPTPSHLNKNPRYLDFRAARRVPESHAWPGLHDHPVVDGGAPGPDAVPVVDLGAADPA
Shenshi33-3   MPTPSHLNKNPRYLDFRAARRVPESHAWPGLHDHPVVDGGAPGPDAVPVVDLGAADPA
PH6WC         MPTPSHLNKNPRYLDFRAARRVPESHAWPGLHDHPVVDGGAPGPDAVPVVDLGAADPA
GY246         MPTPSHLNKNPRYLDFRAARRVPESHAWPGLHDHPVVDGGAPGPDAVPVVDLGAADPA
Lv9kuan       MPTPSHLNKNPRYLDFRAARRVPESHAWPGLHDHPVVDGGAPGPDAVPVVDLGAADPA
Luozao6       MPTPSHLNKNPRYLDFRAARRVPESHAWPGLHDHPVVDGGAPGPDAVPVVDLGAADPA
Chang7-2      MPTPSHLNKNPRYLDFRAARRVPESHAWPGLHDHPVVDGGAPGPDAVPVVDLGAADPA
E28           MPTPSHLNKNPRYLDFRAARRVPESHAWPGLHDHPVVDGGAPGPDAVPVVDLGAADPA
16N125        MPTPSHLNKNPRYLDFRAARRVPESHAWPGLHDHPVVDGGAPGPDAVPVVDLGAADPA
A188          MPTPSHLNKNPRYLDFRAARRVPESHAWPGLHDHPVVDGGAPGPDAVPVVDLGAADPA
Tie7922       MPTPSHLNKNPRYLDFRAARRVPESHAWPGLHDHPVVDGGAPGPDAVPVVDLGAADPA
Liao68        MPTPSHLNKNPRYLDFRAARRVPESHAWPGLHDHPVVDGGAPGPDAVPVVDLGAADPA
B73           MPTPSHLNKNPRYLDFRAARRVPESHAWPGLHDHPVVDGGAPGPDAVPVVDLGAADPA
XL21          MPTPSHLNKNPRYLDFRAARRVPESHAWPGLHDHPVVDGGAPGPDAVPVVDLGAADPA
Liao3162      MPTPSHLNKNPRYLDFRAARRVPESHAWPGLHDHPVVDGGAPGPDAVPVVDLGAADPA
ZX156         MPTPSHLNKNPRYLDFRAARRVPESHAWPGLHDHPVVDGGAPGPDAVPVVDLGAADPA
CML277        MPTPSHLNKNPRYLDFRAARRVPESHAWPGLHDHPVVDGGAPGPDAVPVVDLGAADPA
Ruanerbai     MPTPSHLNKNPRYLDFRAARRVPESHAWPGLHDHPVVDGGAPGPDAVPVVDLGAADPA
```

```
             60        70        80        90        100       110
Si428        PAAAAAVARAAEQWGAFLLTGHGVPADLLARVEDRIATMFALPADDKMRAVRGPGDAC
Dan340       PAAAAAVARAAEQWGAFLLTGHGVPADLLARVEDRIATMFALPADDKMRAVRGPGDAC
Ji853        PAAAAAVARAAEQWGAFLLTGHGVPADLLARVEDRIATMFALPADDKMRAVRGPGDAC
KI3          PAPAAAVARAAEQWGAFLLTGHGVPADLLARVEDRIATMFALPADDKMRAVRGPGDAC
Kangdian11   PAPAAAVARAAEQWGAFLLTGHGVPADLLARVEDRIATMFALPADDKMRAVRGPGDAC
Ye52106      PAPAAAVARAAEQWGAFLLTGHGVPADLLARVEDRIATMFALPADDKMRAVRGPGDAC
Ai34         PAPAAAVARAAEQWGAFLLTGHGVPADLLARVEDRIATMFALPADDKMRAVRGPGDAC
Longkang11   PAAAAAVARAAEQWGAFLLTGHGVPADLLARVEDRIATMFALPADDKMRAVRGPGDAC
Luyuan92     PAAAAAVARAAEQWGAFLLTGHGVPADLLARVEDRIATMFALPADDKMRAVRGPGDAC
C167-1       PAQAAAVARAAEQWGAFLLTGHGVPADLLARVEDRIATMFALPADDKMRAVRGPGDAC
Si444        PAQAAAVARAAEQWGAFLLTGHGVPADLLARVEDRIATMFALPADDKMRAVRGPGDAC
Cheng351     PAQAAAVARAAEQWGAFLLTGHGVPADLLARVEDRIATMFALPADDKMRAVRGPGDAC
142          PAQAAAVARAAEQWGAFLLTGHGVPADLLARVEDRIATMFALPADDKMRAVRGPGDAC
5121         PAQAAAVARAAEQWGAFLLTGHGVPADLLARVEDRIATMFALPADDKMRAVRGPGDAC
OH43         PAQAAAVARAAEQWGAFLLTGHGVPADLLARVEDRIATMFALPADDKMRAVRGPGDAC
1x9801       PAPAAAVARAAEQWGAFLLTGHGVPADLLARVEDRIATMFALPADDKMRAVRGPGDAC
Huangzao4    PAPAAAVARAAEQWGAFLLTGHGVPADLLARVEDRIATMFALPADDKMRAVRGPGDAC
Jun9058      PAQAAAVARAAEQWGAFLLTGHGVPADLLARVEDRIATMFALPADDKMRAVRGPGDAC
598          PAQAAAVARAAEQWGAFLLTGHGVPADLLARVEDRIATMFALPADDKMRAVRGPGDAC
P138         PAQAAAVARAAEQWGAFLLTGHGVPADLLARVEDRIATMFALPADDKMRAVRGPGDAC
Ji69         PAQAAAVARAAEQWGAFLLTGHGVPADLLARVEDRIATMFALPADDKMRAVRGPGDAC
Ke830        PAPAAAVARAAEQWGAFLLTGHGVPADLLARVEDRIATMFALPADDKMRAVRGPGDAC
Kehai181     PAPAAAVARAAEQWGAFLLTGHGVPADLLARVEDRIATMFALPADDKMRAVRGPGDAC
Chang3       PAPAAAVARAAEQWGAFLLTGHGVPADLLARVEDRIATMFALPADDKMRAVRGPGDAC
MO17         PAPAAAVARAAEQWGAFLLTGHGVPADLLARVEDRIATMFALPADDKMRAVRGPGDAC
Sui8941      PAQAAAVARAAEQWGAFLLTGHGVPADLLARVEDRIATMFALPADDKMRAVRGPGDAC
Qi318        PAQAAAVARAAEQWGAFLLTGHGVPADLLARVEDRIATMFALPADDKMRAVRGPGDAC
He344        PAQAAAVARAAEQWGAFLLTGHGVPADLLARVEDRIATMFALPADDKMRAVRGPGDAC
CML460       PAAAAAVARAAEQWGAFLLTGHGVPADLLARVEDRIATMFALPADDKMRAVRGPGDAC
W22          PAAAAAVARAAEQWGAFLLTGHGVPADLLARVEDRIATMFALPADDKMRAVRGPGDAC
PH4CV        PAAAAAVARAAEQWGAFLLTGHGVPADLLARVEDRIATMFALPADDKMRAVRGPGDAC
5237         PAAAAAVARAAEQWGAFLLTGHGVPADLLARVEDRIATMFALPADDKMRAVRGPGDAC
Dan9046      PAAAAAVARAAEQWGAFLLTGHGVPADLLARVEDRIATMFALPADDKMRAVRGPGDAC
4112         PAAAAAVARAAEQWGAFLLTGHGVPADLLARVEDRIATMFALPADDKMRAVRGPGDAC
Shenshi33-3  PAAAAAVARAAEQWGAFLLTGHGVPADLLARVEDRIATMFALPADDKMRAVRGPGDAC
PH6WC        PAAAAAVARAAEQWGAFLLTGHGVPADLLARVEDRIATMFALPADDKMRAVRGPGDAC
GY246        PAAAAAVARAAEQWGAFLLTGHGVPADLLARVEDRIATMFALPADDKMRAVRGPGDAC
Lv9kuan      PAAAAAVARAAEQWGAFLLTGHGVPADLLARVEDRIATMFALPADDKMRAVRGPGDAC
Luozao6      PAAAAAVARAAEQWGAFLLTGHGVPADLLARVEDRIATMFALPADDKMRAVRGPGDAC
Chang7-2     PAAAAAVARAAEQWGAFLLTGHGVPADLLARVEDRIATMFALPADDKMRAVRGPGDAC
E28          PAAAAAVARAAEQWGAFLLTGHGVPADLLARVEDRIATMFALPADDKMRAVRGPGDAC
16N125       PAAAAAVARAAEQWGAFLLTGHGVPADLLARVEDRIATMFALPADDKMRAVRGPGDAC
A188         PAAAAAVARAAEQWGAFLLTGHGVPADLLARVEDRIATMFALPADDKMRAVRGPGDAC
Tie7922      PAPAAAVARAAEQWGAFLLTGHGVPADLLARVEDRIATMFALPADDKMRAVRGPGDAC
Liao68       PAPAAAVARAAEQWGAFLLTGHGVPADLLARVEDRIATMFALPADDKMRAVRGPGDAC
B73          PAPAAAVARAAEQWGAFLLTGHGVPADLLARVEDRIATMFALPADDKMRAVRGPGDAC
XL21         PAPAAAVARAAEQWGAFLLTGHGVPADLLARVEDRIATMFALPADDKMRAVRGPGDAC
Liao3162     PAPAAAVARAAEQWGAFLLTGHGVPADLLARVEDRIATMFALPADDKMRAVRGPGDAC
ZX156        PAPAAAVARAAEQWGAFLLTGHGVPADLLARVEDRIATMFALPADDKMRAVRGPGDAC
CML277       AAPAAAVARAAEQWGAFLLTGHGVPADLLARVEDRIATMFALPADDKMRAVRGPGDAC
Ruanerbai    PAPAAAVARAAEQWGAFLLTGHGVPADLLARVEDRIATMFALPADDKMRAVRGPGDAC
```

	120 130 140 150 160 170
Si428	GYGSPPISSFFSKCMWSEGYTFSPASLRADLRKLWPKAGDDYTSFCDVMEEFHKHMRA
Dan340	GYGSPPISSFFSKCMWSEGYTFSPASLRADLRKLWPKAGDDYTSFCDVMEEFHKHMRA
Ji853	GYGSPPISSFFSKCMWSEGYTFSPASLRADLRKLWPKAGDDYTSFCDVMEEFHKHMRA
KI3	GYGSPPISSFFSKCMWSEGYTFSPASLRADLRKLWPKAGDDYTSFCDVMEEFHKHMRA
Kangdian11	GYGSPPISSFFSKCMWSEGYTFSPASLRADLRKLWPKAGDDYTSFCDVMEEFHKHMRA
Ye52106	GYGSPPISSFFSKCMWSEGYTFSPASLRADLRKLWPKAGDDYTSFCDVMEEFHKHMRA
Ai34	GYGSPPISSFFSKCMWSEGYTFSPASLRADLRKLWPKAGDDYTSFCDVMEEFHKHMRA
Longkang11	GYGSPPISSFFSKCMWSEGYTFSPASLRADLRKLWPKAGDDYTSFCDVMEEFHKHMRA
Luyuan92	GYGSPPISSFFSKCMWSEGYTFSPASLRADLRKLWPKAGDDYTSFCDVMEEFHKHMRA
C167-1	GYGSPPISSFFSKCMWSEGYTFSPASLRADLRKLWPKAGDDYTSFCDVMEEFHKHMRA
Si444	GYGSPPISSFFSKCMWSEGYTFSPASLRADLRKLWPKAGDDYTSFCDVMEEFHKHMRA
Cheng351	GYGSPPISSFFSKCMWSEGYTFSPASLRADLRKLWPKAGDDYTSFCDVMEEFHKHMRA
142	GYGSPPISSFFSKCMWSEGYTFSPASLRADLRKLWPKAGDDYTSFCDVMEEFHKHMRA
5121	GYGSPPISSFFSKCMWSEGYTFSPASLRADLRKLWPKAGDDYTSFCDVMEEFHKHMRA
OH43	GYGSPPISSFFSKCMWSEGYTFSPASLRADLRKLWPKAGDDYTSFCDVMEEFHKHMRA
lx9801	GYGSPPISSFFSKCMWSEGYTFSPASLRADLRKLWPKAGDDYTSFCDVMEEFHKHMRA
Huangzao4	GYGSPPISSFFSKCMWSEGYTFSPASLRADLRKLWPKAGDDYTSFCDVMEEFHKHMRA
Jun9058	GYGSPPISSFFSKCMWSEGYTFSPASLRADLRKLWPKAGDDYTSFCDVMEEFHKHMRA
598	GYGSPPISSFFSKCMWSEGYTFSPASLRADLRKLWPKAGDDYTSFCDVMEEFHKHMRA
P138	GYGSPPISSFFSKCMWSEGYTFSPASLRADLRKLWPKAGDDYTSFCDVMEEFHKHMRA
Ji69	GYGSPPISSFFSKCMWSEGYTFSPASLRADLRKLWPKAGDDYTSFCDVMEEFHKHMRA
Ke830	GYGSPPISSFFSKCMWSEGYTFSPASLRADLRKLWPKAGDDYTSFCDVMEEFHKHMRA
Kehai181	GYGSPPISSFFSKCMWSEGYTFSPASLRADLRKLWPKAGDDYTSFCDVMEEFHKHMRA
Chang3	GYGSPPISSFFSKCMWSEGYTFSPASLRADLRKLWPKAGDDYTSFCDVMEEFHKHMRA
MO17	GYGSPPISSFFSKCMWSEGYTFSPASLRADLRKLWPKAGDDYTSFCDVMEEFHKHMRA
Sui8941	GYGSPPISSFFSKCMWSEGYTFSPASLRADLRKLWPKAGDDYTSFCDVMEEFHKHMRA
Qi318	GYGSPPISSFFSKCMWSEGYTFSPASLRADLRKLWPKAGDDYTSFCDVMEEFHKHMRA
He344	GYGSPPISSFFSKCMWSEGYTFSPASLRADLRKLWPKAGDDYTSFCDVMEEFHKHMRA
CML460	GYGSPPISSFFSKCMWSEGYTFSPASLRADLRKLWPKAGDDYTSFCDVMEEFHKHMRA
W22	GYGSPPISSFFSKCMWSEGYTFSPASLRADLRKLWPKAGDDYTSFCDVMEEFHKHMRA
PH4CV	GYGSPPISSFFSKCMWSEGYTFSPASLRADLRKLWPKAGDDYTSFCDVMEEFHKHMRA
5237	GYGSPPISSFFSKCMWSEGYTFSPASLRADLRKLWPKAGDDYTSFCDVMEEFHKHMRA
Dan9046	GYGSPPISSFFSKCMWSEGYTFSPASLRADLRKLWPKAGDDYTSFCDVMEEFHKHMRA
4112	GYGSPPISSFFSKCMWSEGYTFSPASLRADLRKLWPKAGDDYTSFCDVMEEFHKHMRA
Shenshi33-3	GYGSPPISSFFSKCMWSEGYTFSPASLRADLRKLWPKAGDDYTSFCDVMEEFHKHMRA
PH6WC	GYGSPPISSFFSKCMWSEGYTFSPASLRADLRKLWPKAGDDYTSFCDVMEEFHKHMRA
GY246	GYGSPPISSFFSKCMWSEGYTFSPASLRADLRKLWPKAGDDYTSFCDVMEEFHKHMRA
Lv9kuan	GYGSPPISSFFSKCMWSEGYTFSPASLRADLRKLWPKAGDDYTSFCDVMEEFHKHMRA
Luozao6	GYGSPPISSFFSKCMWSEGYTFSPASLRADLRKLWPKAGDDYTSFCDVMEEFHKHMRA
Chang7-2	GYGSPPISSFFSKCMWSEGYTFSPASLRADLRKLWPKAGDDYTSFCDVMEEFHKHMRA
E28	GYGSPPISSFFSKCMWSEGYTFSPASLRADLRKLWPKAGDDYTSFCDVMEEFHKHMRA
16N125	GYGSPPNSSFFSKCMWSEGYTFSPASLRADLRKLWPKAGDDYTSFCDVMEEFHKHMRA
A188	GYGSPPISSFFSKCMWSEGYTFSPASLRADLRKLWPKAGDDYTSFCDVMEEFHKHMRA
Tie7922	GYGSPPISSFFSKCMWSEGYTFSPASLRADLRKLWPKAGDDYTSFCDVMEEFHKHMRA
Liao68	GYGSPPISSFFSKCMWSEGYTFSPASLRADLRKLWPKAGDDYTSFCDVMEEFHKHMRA
B73	GYGSPPISSFFSKCMWSEGYTFSPASLRADLRKLWPKAGDDYTSFCDVMEEFHKHMRA
XL21	GYGSPPISSFFSKCMWSEGYTFSPASLRADLRKLWPKAGDDYTSFCDVMEEFHKHMRA
Liao3162	GYGSPPISSFFSKCMWSEGYTFSPASLRADLRKLWPKAGDDYTSFCDVMEEFHKHMRA
ZX156	GYGSPPISSFFSKCMWSEGYTFSPASLRADLRKLWPKAGDDYTSFCDVMEEFHKHMRA
CML277	GYGSPPISSFFSKCMWSEGYTFSPASLRADLRKLWPKAGDDYTSFCDVMEEFHKHMRA
Ruanerbai	GYGSPPISSFFSKCMWSEGYTFSPASLRADLRKLWPKAGDDYTSFCDVMEEFHKHMRA

```
                     180       190       200       210       220       230
Si428          LADKLLELFLMALGLTDEQASAVEAERRIAETMTATMHLNWYPRCPDPRRALGLIAHT
Dan340         LADKLLELFLMALGLTDEQASAVEAERRIAETMTATMHLNWYPRCPDPRRALGLIAHT
Ji853          LADKLLELFLMALGLTDEQASAVEAERRIAETMTATMHLNWYPRCPDPRRALGLIAHT
KI3            LADKLLELFLMALGLTDEQASAVEAERRIAETMTATMHLNWYPRCPDPRRALGLIAHT
Kangdian11     LADKLLELFLMALGLTDEQASAVEAERRIAETMTATMHLNWYPRCPDPRRALGLIAHT
Ye52106        LADKLLELFLMALGLTDEQASAVEAERRIAETMTATMHLNWYPRCPDPRRALGLIAHT
Ai34           LADKLLELFLMALGLTDEQASAVEAERRIAETMTATMHLNWYPRCPDPRRALGLIAHT
Longkang11     LADKLLELFLMALGLTDEQASAVEAERRIAETMTATMHLNWYPRCPDPRRALGLIAHT
Luyuan92       LADKLLELFLMALGLTDEQASAVEAERRIAETMTATMHLNWYPRCPDPRRALGLIAHT
C167-1         LADKLLELFLMALGLTDEQASAVEAERRIAETMTATMHLNWYPRCPDPRRALGLIAHT
Si444          LADKLLELFLMALGLTDEQASAVEAERRIAETMTATMHLNWYPRCPDPRRALGLIAHT
Cheng351       LADKLLELFLMALGLTDEQASAVEAERRIAETMTATMHLNWYPRCPDPRRALGLIAHT
142            LADKLLELFLMALGLTDEQASAVEAERRIAETMTATMHLNWYPRCPDPRRALGLIAHT
5121           LADKLLELFLMALGLTDEQASAVEAERRIAETMTATMHLNWYPRCPDPRRALGLIAHT
OH43           LADKLLELFLMALGLTDEQASAVEAERRIAETMTATMHLNWYPRCPDPRRALGLIAHT
lx9801         LADKLLELFLMALGLTDEQASAVEAERRIAETMTATMHLNWYPRCPDPRRALGLIAHT
Huangzao4      LADKLLELFLMALGLTDEQASAVEAERRIAETMTATMHLNWYPRCPDPRRALGLIAHT
Jun9058        LADKLLELFLMALGLTDEQASAVEAERRIAETMTATMHLNWYPRCPDPRRALGLIAHT
598            LADKLLELFLMALGLTDEQASAVEAERRIAETMTATMHLNWYPRCPDPRRALGLIAHT
P138           LADKLLELFLMALGLTDEQASAVEAERRIAETMTATMHLNWYPRCPDPRRALGLIAHT
Ji69           LADKLLELFLMALGLTDEQASAVEAERRIAETMTATMHLNWYPRCPDPRRALGLIAHT
Ke830          LADKLLELFLMALGLTDEQASAVEAERRIAETMTATMHLNWYPRCPDPRRALGLIAHT
Kehai181       LADKLLELFLMALGLTDEQASAVEAERRIAETMTATMHLNWYPRCPDPRRALGLIAHT
Chang3         LADKLLELFLMALGLTDEQASAVEAERRIAETMTATMHLNWYPRCPDPRRALGLIAHT
MO17           LADKLLELFLMALGLTDEQASAVEAERRIAETMTATMHLNWYPRCPDPRRALGLIAHT
Sui8941        LADKLLELFLMALGLTDEQASAVEAERRIAETMTATMHLNWYPRCPDPRRALGLIAHT
Qi318          LADKLLELFLMALGLTDEQASAVEAERRIAETMTATMHLNWYPRCPDPRRALGLIAHT
He344          LADKLLELFLMALGLTDEQASAVEAERRIAETMTATMHLNWYPRCPDPRRALGLIAHT
CML460         LADKLLELFLMALGLTDEQASAVEAERRIAETMTATMHLNWYPRCPDPRRALGLIAHT
W22            LADKLLELFLMALGLTDEQASAVEAERRIAETMTATMHLNWYPRCPDPRRALGLIAHT
PH4CV          LADKLLELFLMALGLTDEQASAVEAERRIAETMTATMHLNWYPRCPDPRRALGLIAHT
5237           LADKLLELFLMALGLTDEQASAVEAERRIAETMTATMHLNWYPRCPDPRRALGLIAHT
Dan9046        LADKLLELFLMALGLTDEQASAVEAERRIAETMTATMHLNWYPRCPDPRRALGLIAHT
4112           LADKLLELFLMALGLTDEQASAVEAERRIAETMTATMHLNWYPRCPDPRRALGLIAHT
Shenshi33-3    LADKLLELFLMALGLTDEQASAVEAERRIAETMTATMHLNWYPRCPDPRRALGLIAHT
PH6WC          LADKLLELFLMALGLTDEQASAVEAERRIAETMTATMHLNWYPRCPDPRRALGLIAHT
GY246          LADKLLELFLMALGLTDEQASAVEAERRIAETMTATMHLNWYPRCPDPRRALGLIAHT
Lv9kuan        LADKLLELFLMALGLTDEQASAVEAERRIAETMTATMHLNWYPRCPDPRRALGLIAHT
Luozao6        LADKLLELFLMALGLTDEQASAVEAERRIAETMTATMHLNWYPRCPDPRRALGLIAHT
Chang7-2       LADKLLELFLMALGLTDEQASAVEAERRIAETMTATMHLNWYPRCPDPRRALGLIAHT
E28            LADKLLELFLMALGLTDEQASAVEAERRIAETMTATMHLNWYPRCPDPRRALGLIAHT
16N125         LADKLLELFLMALGLTDEQASAVEAERRIAETMTATMHLNWYPRCPDPRRALGLIAHT
A188           LADKLLELFLMALGLTDEQASAVEAERRIAETMTATMHLNWYPRCPDPRRALGLIAHT
Tie7922        LADKLLELFLMALGLTDEQASAVEAERRIAETMTATMHLNWYPRCPDPRRALGLIAHT
Liao68         LADKLLELFLMALGLTDEQASAVEAERRIAETMTATMHLNWYPRCPDPRRALGLIAHT
B73            LADKLLELFLMALGLTDEQASAVEAERRIAETMTATMHLNWYPRCPDPRRALGLIAHT
XL21           LADKLLELFLMALGLTDEQASAVEAERRIAETMTATMHLNWYPRCPDPRRALGLIAHT
Liao3162       LADKLLELFLMALGLTDEQASAVEAERRIAETMTATMHLNWYPRCPDPRRALGLIAHT
ZX156          LADKLLELFLMALGLTDEQASAVEAERRIAETMTATMHLNWYPRCPDPRRALGLIAHT
CML277         LADKLLELFLMALGLTDEQASAVEAERRIAETMTATMHLNWYPRCPDPRRALGLIAHT
Ruanerbai      LADKLLELFLMALGLTDEQASAVEAERRIAETMTATMHLNWYPRCPDPRGALGLIAHT
```

```
                          240       250       260       270       280       290
Si428          DSGFFTFVMQSLVPGLQLFRHAPDRWVAVPAVPGAFVVNVGDLFHILTNGRFHSVYHR
Dan340         DSGFFTFVMQSLVPGLQLFRHAPDRWVAVPAVPGAFVVNVGDLFHILTNGRFHSVYHR
Ji853          DSGFFTFVMQSLVPGLQLFRHAPDRWVAVPAVPGAFVVNVGDLFHILTNGRFHSVYHR
KI3            DSGFFTFVMQSLVPGLQLFRHAPDRWVAVPAVPGAFVVNVGDLFHILTNGRFHSVYHR
Kangdian11     DSGFFTFVMQSLVPGLQLFRHAPDRWVAVPAVPGAFVVNVGDLFHILTNGRFHSVYHR
Ye52106        DSGFFTFVMQSLVPGLQLFRHAPDRWVAVPAVPGAFVVNVGDLFHILTNGRFHSVYHR
Ai34           DSGFFTFVMQSLVPGLQLFRHAPDRWVAVPAVPGAFVVNVGDLFHILTNGRFHSVYHR
Longkang11     DSGFFTFVMQSLVPGLQLFRHAPDRWVAVPAAPGAFVVNVGDLFHILTNGRFHSVYHR
Luyuan92       DSGFFTFVMQSLVPGLQLFRHAPDRWVAVPAAPGAFVVNVGDLFHILTNGRFHSVYHR
C167-1         DSGFFTFVMQSLVPGLQLFRHAPDRWVAVPAAPGAFVVNVGDLFHILTNGRFHSVYHR
Si444          DSGFFTFVMQSLVPGLQLFRHAPDRWVAVPAAPGAFVVNVGDLFHILTNGRFHSVYHR
Cheng351       DSGFFTFVMQSLVPGLQLFRHAPDRWVAVPAAPGAFVVNVGDLFHILTNGRFHSVYHR
142            DSGFFTFVMQSLVPGLQLFRHAPDRWVAVPAAPGAFVVNVGDLFHILTNGRFHSVYHR
5121           DSGFFTFVMQSLVPGLQLFRHAPDRWVAVPAAPGAFVVNVGDLFHILTNGRFHSVYHR
OH43           DSGFFTFVMQSLVPGLQLFRHAPDRWVAVPAAPGAFVVNVGDLFHILTNGRFHSVYHR
lx9801         DSGFFTFVMQSLVPGLQLFRHAPDRWVAVPAVPGAFVVNVGDLFHILTNGRFHSVYHR
Huangzao4      DSGFFTFVMQSLVPGLQLFRHAPDRWVAVPAVPGAFVVNVGDLFHILTNGRFHSVYHR
Jun9058        DSGFFTFVMQSLVPGLQLFRHAPDRWVSVPAVPGAFVVNVGDLFHILTNGRFHSVYHR
598            DSGFFTFVMQSLVPGLQLFRHAPDRWVSVPAVPGAFVVNVGDLFHILTNGRFHSVYHR
P138           DSGFFTFVMQSLVPGLQLFRHAPDRWVSVPAVPGAFVVNVGDLFHILTNGRFHSVYHR
Ji69           DSGFFTFVMQSLVPGLQLFRHAPDRWVAVPAVPGAFVVNVGDLFHILTNGRFHSVYHR
Ke830          DSGFFTFVMQSLVPGLQLFRHAPDRWVAVPAAPGAFVVNVGDLFHILTNGRFHSVYHR
Kehai181       DSGFFTFVMQSLVPGLQLFRHAPDRWVAVPAAPGAFVVNVGDLFHILTNGRFHSVYHR
Chang3         DSGFFTFVMQSLVPGLQLFRHAPDRWVAVPAAPGAFVVNVGDLFHILTNGRFHSVYHR
MO17           DSGFFTFVMQSLVPGLQLFRHAPDRWVAVPAAPGAFVVNVGDLFHILTNGRFHSVYHR
Sui8941        DSGFFTFVMQSLVPGLQLFRHAPDRWVAVPAAPGAFVVNVGDLFHILTNGRFHSVYHR
Qi318          DSGFFTFVMQSLVPGLQLFRHAPDRWVAVPAAPGAFVVNVGDLFHILTNGRFHSVYHR
He344          DSGFFTFVMQSLVPGLQLFRHAPDRWVAVPAAPGAFVVNVGDLFHILTNGRFHSVYHR
CML460         DSGFFTFVMQSLVPGLQLFRHAPDRWVAVPAVPGAFVVNVGDLFHILTNGRFHSVYHR
W22            DSGFFTFVMQSLVPGLQLFRHAPDRWVAVPAVPGAFVVNVGDLFHILTNGRFHSVYHR
PH4CV          DSGFFTFVMQSLVPGLQLFRHAPDRWVAVPAVPGAFVVNVGDLFHILTNGRFHSVYHR
5237           DSGFFTFVMQSLVPGLQLFRHAPDRWVAVPAVPGAFVVNVGDLFHILTNGRFHSVYHR
Dan9046        DSGFFTFVMQSLVPGLQLFRHAPDRWVAVPAVPGAFVVNVGDLFHILTNGRFHSVYHR
4112           DSGFFTFVMQSLVPGLQLFRHAPDRWVAVPAVPGAFVVNVGDLFHILTNGRFHSVYHR
Shenshi33-3    DSGFFTFVMQSLVPGLQLFRHAPDRWVAVPAVPGAFVVNVGDLFHILTNGRFHSVYHR
PH6WC          DSGFFTFVMQSLVPGLQLFRHAPDRWVAVPAVPGAFVVNVGDLFHILTNGRFHSVYHR
GY246          DSGFFTFVMQSLVPGLQLFRHAPDRWVAVPAVPGAFVVNVGDLFHILTNGRFHSVYHR
Lv9kuan        DSGFFTFVMQSLVPGLQLFRHAPDRWVAVPAVPGAFVVNVGDLFHILTNGRFHSVYHR
Luozao6        DSGFFTFVMQSLVPGLQLFRHAPDRWVAVPAVPGAFVVNVGDLFHILTNGRFHSVYHR
Chang7-2       DSGFFTFVMQSLVPGLQLFRHAPDRWVAVPAVPGAFVVNVGDLFHILTNGRFHSVYHR
E28            DSGFFTFVMQSLVPGLQLFRHAPDRWVAVPAVPGAFVVNVGDLFHILTNGRFHSVYHR
16N125         DSGFFTFVMQSLVPGLQLFRHAPDRWVAVPAVPGAFVVNVGDLFHILTNGRFHSVYHR
A188           DSGFFTFVMQSLVPGLQLFRHAPDRWVAVPAVPGAFVVNVGDLFHILTNGRFHSVYHR
Tie7922        DSGFFTFVMQSLVPGLQLFRHAPDRWVAVPAVPGAFVVNVGDLFHILTNGRFHSVYHR
Liao68         DSGFFTFVMQSLVPGLQLFRHAPDRWVAVPAVPGAFVVNVGDLFHILTNGRFHSVYHR
B73            DSGFFTFVMQSLVPGLQLFRHAPDRWVAVPAVPGAFVVNVGDLFHILTNGRFHSVYHR
XL21           DSGFFTFVMQSLVPGLQLFRHAPDRWVAVPAVPGAFVVNVGDLFHILTNGRFHSVYHR
Liao3162       DSGFFTFVMQSLVPGLQLFRHAPDRWVAVPAVPGAFVVNVGDLFHILTNGRFHSVYHR
ZX156          DSGFFTFVMQSLVPGLQLFRHAPDRWVAVPAVPGAFVVNVGDLFHILTNGRFHSVYHR
CML277         DSGFFTFVMQSLVPGLQLFRHAPDRWVAVPAVPGAFVVNVGDLFHILTNGRFHSVYHR
Ruanerbai      DSGFFAFEMQSLVAGLQLFRHAPDRWVAVPAVPGAFVVNVGDVFHILTNGRFHSVYHR
```

```
                     300       310       320       330       340
Si428        AVVNRDLDRISLGYFLGPPPHAKVAPLREAVPPGRAPAYRAVTWPEYMGVRKKAFTTG
Dan340       AVVNRDLDRISLGYFLGPPPHAKVAPLREAVPPGRAPAYRAVTWPEYMGVRKKAFTTG
Ji853        AVVNRDLDRISLGYFLGPPPHAKVAPLREAVPPGRAPAYRAVTWPEYMGVRKKAFTTG
KI3          AVVNRDLDRISLGYFLGPPPHAKVAPLREAVPPGRAPAYRAVTWPEYMGVRKKAFTTG
Kangdian11   AVVNRDLDRISLGYFLGPPPHAKVAPLREAVPPGRAPAYRAVTWPEYMGVRKKAFTTG
Ye52106      AVVNRDLDRISLGYFLGPPPHAKVAPLREAVPPGRAPAYRAVTWPEYMGVRKKAFTTG
Ai34         AVVNRDLDRISLGYFLGPPPHAKVAPLREAVPPGRAPAYRAVTWPEYMGVRKKAFTTG
Longkang11   AVVNRDLDRISLGYFLGPPPHAKVAPLREAVPPGRAPAYRAVTWPEYMGVRKKAFTTG
Luyuan92     AVVNRDLDRISLGYFLGPPPHAKVAPLREAVPPGRAPAYRAVTWPEYMGVRKKAFTTG
C167-1       AVVNRDLDRISLGYFLGPPPHAKVAPLREAVPPGRAPAYRAVTWPEYMGVRKKAFTTG
Si444        AVVNRDLDRISLGYFLGPPPHAKVAPLREAVPPGRAPAYRAVTWPEYMGVRKKAFTTG
Cheng351     AVVNRDLDRISLGYFLGPPPHAKVAPLREAVPPGRAPAYRAVTWPEYMGVRKKAFTTG
142          AVVNRDLDRISLGYFLGPPPHAKVAPLREAVPPGRAPAYRAVTWPEYMGVRKKAFTTG
5121         AVVNRDLDRISLGYFLGPPPHAKVAPLREAVPPGRAPAYRAVTWPEYMGVRKKAFTTG
OH43         AVVNRDLDRISLGYFLGPPPHAKVAPLREAVPPGRAPAYRAVTWPEYMGVRKKAFTTG
lx9801       AVVNRDLDRISLGYFLGPPPHAKVAPLREAVPPGRAPAYRAVTWPEYMGVRKKAFTTG
Huangzao4    AVVNRDLDRISLGYFLGPPPHAKVAPLREAVPPGRAPAYRAVTWPEYMGVRKKAFTTG
Jun9058      AVVNRDLDRISLGYFLGPPPHAKVAPLREAVPPGRAPAYRAVTWPEYMGVRKKAFTTG
598          AVVNRDLDRISLGYFLGPPPHAKVAPLREAVPPGRAPAYRAVTWPEYMGVRKKAFTTG
P138         AVVNRDLDRISLGYFLGPPPHAKVAPLREAVPPGRAPAYRAVTWPEYMGVRKKAFTTG
Ji69         AVVNRDLDRISLGYFLGPPPHAKVAPLREAVPPGRAPAYRAVTWPEYMGVRKKAFTTG
Ke830        AVVNRDLDRISLGYFLGPPPHAKVAPLREAVPPGRAPAYRAVTWPEYMGVRKKAFTTG
Kehai181     AVVNRDLDRISLGYFLGPPPHAKVAPLREAVPPGRAPAYRAVTWPEYMGVRKKAFTTG
Chang3       AVVNRDLDRISLGYFLGPPPHAKVAPLREAVPPGRAPAYRAVTWPEYMGVRKKAFTTG
MO17         AVVNRDLDRISLGYFLGPPPHAKVAPLREAVPPGRAPAYRAVTWPEYMGVRKKAFTTG
Sui8941      AVVNRDLDRISLGYFLGPPPHAKVAPLREAVPPGRAPAYRAVTWPEYMGVRKKAFTTG
Qi318        AVVNRDHDRISLGYFLGPPPHAKVAPLREAVPPGRAPAYRAVTWPEYMGVRKKAFTTG
He344        AVVNRDLDRISLGYFLGPPPHAKVAPLREAVPPGRAPAYRAVTWPEYMGVRKKAFTTG
CML460       AVVNRDLDRISLGYFLGPPPHAKVAPLREAVPPGRAPAYRAVTWPEYMGVRKKAFTTG
W22          AVVNRDLDRISLGYFLGPPPHAKVAPLREAVPPGRAPAYRAVTWPEYMGVRKKAFTTG
PH4CV        AVVNRDLDRISLGYFLGPPPHAKVAPLREAVPPGRAPAYRAVTWPEYMGVRKKAFTTG
5237         AVVNRDLDRISLGYFLGPPPHAKVAPLREAVPPGRAPAYRAVTWPEYMGVRKKAFTTG
Dan9046      AVVNRDLDRISLGYFLGPPPHAKVAPLREAVPPGRAPAYRAVTWPEYMGVRKKAFTTG
4112         AVVNRDLDRISLGYFLGPPPHAKVAPLREAVPPGRAPAYRAVTWPEYMGVRKKAFTTG
Shenshi33-3  AVVNRDLDRISLGYFLGPPPHAKVAPLREAVPPGRAPAYRAVTWPEYMGVRKKAFTTG
PH6WC        AVVNRDLDRISLGYFLGPPPHAKVAPLREAVPPGRAPAYRAVTWPEYMGVRKKAFTTG
GY246        AVVNRDLDRISLGYFLGPPPHAKVAPLREAVPPGRAPAYRAVTWPEYMGVRKKAFTTG
Lv9kuan      AVVNRDLDRISLGYFLGPPPHAKVAPLREAVPPGRAPAYRAVTWPEYMGVRKKAFTTG
Luozao6      AVVNRDLDRISLGYFLGPPPHAKVAPLREAVPPGRAPAYRAVTWPEYMGVRKKAFTTG
Chang7-2     AVVNRDLDRISLGYFLGPPPHAKVAPLREAVPPGRAPAYRAVTWPEYMGVRKKAFTTG
E28          AVVNRDLDRISLGYFLGPPPHAKVAPLREAVPPGRAPAYRAVTWPEYMGVRKKAFTTG
16N125       AVVNRDLDRISLGYFLGPPPHAKVAPLREAVPPGRAPAYRAVTWPEYMGVRKKAFTTG
A188         AVVNRDLDRISLGYFLGPPPHAKVAPLREAVPPGRAPAYRAVTWPEYMGVRKKAFTTG
Tie7922      AVVNRDLDRISLGYFLGPPPHAKVAPLREAVPPGRAPAYRAVTWPEYMGVRKKAFTTG
Liao68       AVVNRDLDRISLGYFLGPPPHAKVAPLREAVPPGRAPAYRAVTWPEYMGVRKKAFTTG
B73          AVVNRDLDRISLGYFLGPPPHAKVAPLREAVPPGRAPAYRAVTWPEYMGVRKKAFTTG
XL21         AVVNRDLDRISLGYFLGPPPHAKVAPLREAVPPGRAPAYRAVTWPEYMGVRKKAFTTG
Liao3162     AVVNRDLDRISLGYFLGPPPHAKVAPLREAVPPGRAPAYRAVTWPEYMGVRKKAFTTG
ZX156        AVVNRDLDRISLGYFLGPPPHAKVAPLREAVPPGRAPAYRAVTWPEYMGVRKKAFTTG
CML277       AVVNRDLDRISLGYFLGPPPHAKVAPLREAVPPGRAPAYRAVTWPEYMGVRKKAFTTG
Ruanerbai    AVVNRDLDRISLGYFVGPPPHAKVAPLREAVPPGRAPAYRAVTWPEYMGVRKKAFTTG
```

```
                350       360       370       380
                  .         .         .         .
Si428           ASALKMVALAAAADLDDGDAAAADPAVVHQQQQQQLVVSS..
Dan340          ASALKMVALAAAADLDDGDAAAADPAVVHQQQQQQLVVSS..
Ji853           ASALKMVALAAAADLDDGDAAAADPAVVHQQQQQQLVVSS..
KI3             ASALKMVALAAAADLDDGDAAAADPAVVHQQQQQQLVVSS..
Kangdian11      ASALKMVALAAAAADLDD.GDAAAADPAVVHQQQQLVVSS..
Ye52106         ASALKMVALAAAAADLDD.GDAAAADPAVVHQQQQLVVSS..
Ai34            ASALKMVALAAAAADLDD.GDAAAADPAVVHQQQQLVVSS..
Longkang11      ASALKMVALAAAAAADLDDGDAAAADPAVVHQQQQLVVSS..
Luyuan92        ASALKMVALAAAAAADLDDGDAAAADPAVVHQQQQLVVSS..
C167-1          ASALKMVALAAAAAADLDDGDAAAADPAVVHQQQQLVVSS..
Si444           ASALKMVALAAAAAADLDDGDAAAADPAVVHQQQQLVVSS..
Cheng351        ASALKMVALAAAAAADLDDGDAAAADPAVVHQQQQLVVSS..
142             ASALKMVALAAAAAADLDDGDAAAADPAVVHQQQQLVVSS..
5121            ASALKMVALAAAAAADLDDGDAAAADPAVVHQQQQLVVSS..
OH43            ASALKMVALAAAAAADLDDGDAAAADPAVVHQQQQLVVSS..
lx9801          ASALKMVALAAAAAADLDDGDAAAADPAVVHQQQQLVVSS..
Huangzao4       ASALKMVALAAAAAADLDDGDAAAADPAVVHQQQQLVVSS..
Jun9058         ASALKMVALAAAADLDDD......GDAAVVHQQQQLVVSS..
598             ASALKMVALAAAADLDDD......GDAAVVHQQQQLVVSS..
P138            ASALKMVALAAAADLDDD......GDAAVVHQQQQLVVSS..
Ji69            ASALKMVALAAAADLDDD......GDAAVVHQQQQLVVSS..
Ke830           ASALKMVALAAAADLDDD......GDAAVVHQQQQLVVSS..
Kehai181        ASALKMVALAAAADLDDD......GDAAVVHQQQQLVVSS..
Chang3          ASALKMVALAAAADLDDD......GDAAVVHQQQQLVVSS..
MO17            ASALKMVALAAAADLDDD......GDAAVVHQQQQLVVSS..
Sui8941         ASALKMVALAAAADLDDD......GDAAVVHQQQQLVVSS..
Qi318           ASALKMVALAAAADLDDD......GDAAVVHQQQQLVVSS..
He344           ASALKMVALAAAADLDDD......GDAAVVHQQQQLVVSS..
CML460          ASALKMVALAAAADLDDD......GDAAVVHQQQQLVVSS..
W22             ASALKMVALAAAADLDDD......GDAAVVHQQQQLVVSS..
PH4CV           ASALKMVALAAAADLDDD......GDAAVVHQQQQLVVSS..
5237            ASALKMVALAAAADLDDD......GDAAVVHQQQQLVVSS..
Dan9046         ASALKMVALAAAADLDDD......GDAAVVHQQQQLVVSS..
4112            ASALKMVALAAAADLDDD......GDAAVVHQQQQLVVSS..
Shenshi33-3     ASALKMVALAAAADLDDD......GDAAVVHQQQQLVVSS..
PH6WC           ASALKMVALAAAADLDDD......GDAAVVHQQQQLVVSS..
GY246           ASALKMVALAAAADLDDD......GDAAVVHQQQQLVVSS..
Lv9kuan         ASALKMVALAAAADLDDD......GDAAVVHQQQQLVVSS..
Luozao6         ASALKMVALAAAADLDDD......GDAAVVHQQQQLVVSS..
Chang7-2        ASALKMVALAAAADLDDD......GDAAVVHQQQQLVVSS..
E28             ASALKMVALAAAADLDDD......GDAAVVHQQQQLVVSS..
16N125          ASALKMVALAAAADLDDD......GDAAVVHQQQQLVVSS..
A188            ASALKMVALAAAADLDDD......GDAAVVHQQQQQQLVVSS
Tie7922         ASALKMVALAAAADLDDD......GDAAVVHQQQQLVVSS..
Liao68          ASALKMVALAAAADLDDD......GDAAVVHQQQQLVVSS..
B73             ASALKMVALAAAADLDDD......GDAAVVHQQQQLVVSS..
XL21            ASALKMVALAAAADLDDD......GDAAVVHQQQQLVVSS..
Liao3162        ASALKMVALAAAADLDDD......GDAAVVHQQQQLVVSS..
ZX156           ASALKMVALAAAADLDDD......GDAAVVHQQQQLVVSS..
CML277          ASALKMVALAAAADLDDD......GDAAVVHQQQQLVVSS..
Ruanerbai       ASALKMVALAAVADLDDGDAAG..ADPAVVHQQQLVVSS...
```
